# New Hosts and Records in Portugal for the Root-Knot Nematode *Meloidogyne luci*

**DOI:** 10.21307/jofnem-2019-003

**Published:** 2019-06-04

**Authors:** Duarte Santos, António Correia, Isabel Abrantes, Carla Maleita

**Affiliations:** 1 CFE – Centre for Functional Ecology-Science for People & the Planet, Department of Life Sciences, University of Coimbra (UC), Calçada Martim de Freitas, P-3000 456, Coimbra, Portugal; 2 CIEPQPF – Chemical Process Engineering and Forest Products Research Centre, Department of Chemical Engineering, UC, P-3030 790, Coimbra, Portugal

**Keywords:** *Cordyline australis*, Esterase phenotype, Molecular biology, *Oxalis corniculata*, Plant-parasitic nematodes

**Figure 1:**
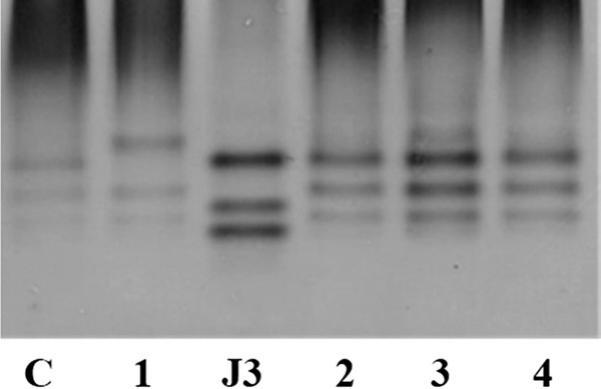
Esterase phenotypes of protein homogenates from five egg-laying females of *Meloidogyne* species isolates. C – *M. luci* (positive control); 1 – *M. ethiopica* isolate from Brazil; J3 – *M. javanica* (reference isolate); 2 – *M. luci* (*Cordyline australis*); 3 – *M. luci* (*Solanum lycopersicum*) and 4 – *M. luci* (*Oxalis corniculata*).

**Figure 2:**
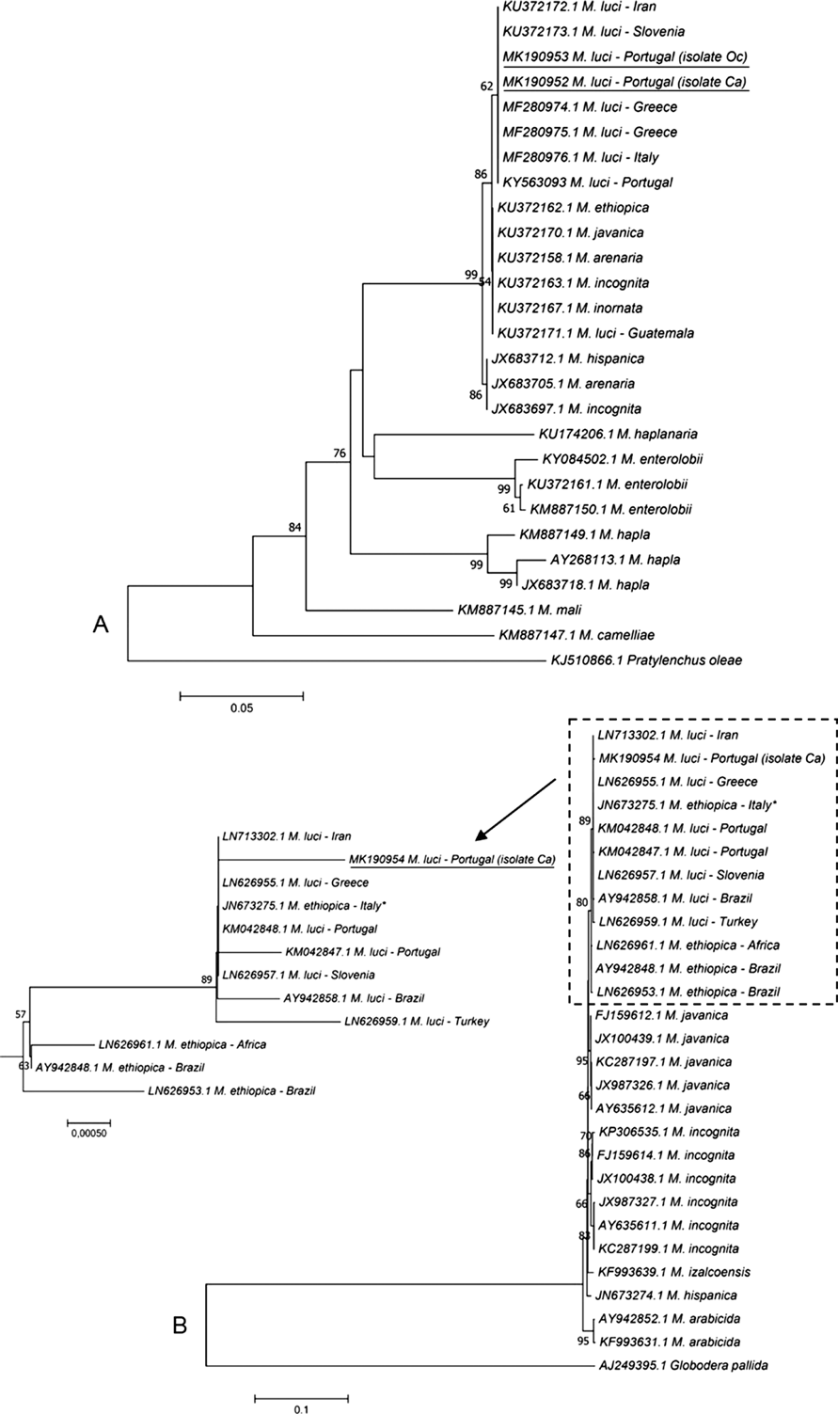
Phylogenetic relationship of *Meloidogyne* spp. sequences based on the alignment of sequences of cytochrome oxidase subunit I (A) and cytochrome oxidase subunit II (B) of mitochondrial DNA with available sequences of other *Meloidogyne* species. The phylogenetic tree was generated in MEGA7 using the Neighbour-Joining method. The percentage of replicate trees in which the associated *Meloidogyne* spp. clustered together in the bootstrap test (500 replicates) is shown next to the branches. The evolutionary distances were computed using the Jukes-Cantor method. All positions with less than 95% site coverage were eliminated. That is, fewer than 5% alignment gaps, missing data, and ambiguous bases were allowed at any position. *Recently, reclassified as *M. luci*, according to Stare et al. (2017).

Several species of the genus *Meloidogyne* (root-knot nematodes, RKN) have been reported in Portugal: *M. arenaria* (Neal, 1889) Chitwood, 1949; *M. chitwoodi* Golden et al., 1980; *M. hapla* Chitwood, 1949; *M. hispanica* Hirschmann, 1986; *M. incognita* (Kofoid and White, 1919) Chitwood, 1949; *M. javanica* (Treub, 1885) Chitwood, 1949; and *M. lusitanica* Abrantes and Santos, 1991 (Abrantes et al., 2008; Conceição et al., 2009). In 2013, the tropical RKN, *M. luci* Carneiro et al., 2014, was detected in a potato field near Coimbra, Portugal (Maleita et al., 2018). *Meloidogyne luci*, added to the European Plant Protection Organization Alert List in 2017, was also found parasitizing maize (*Zea mays* L.) and kiwi (*Actinidia* spp.) in Greece; and tomato (*Solanum lycopersicum* L.) in Italy and Slovenia (Širca et al., 2004; Conceição et al., 2012; Maleita et al., 2012). In Brazil, Iran, Chile, Guatemala, and Turkey, *M. luci* has been found associated with several important vegetable plants and fruit tree species (Aydınlı et al., 2013; Carneiro et al., 2014; Bellé et al., 2016; Janssen et al., 2016; Machado et al., 2016). Currently, there are about 26 different plant species recognized as hosts for *M. luci* (EPPO, 2017). Because of its morphological resemblance to *M. ethiopica* Whitehead, 1968 and similar esterase phenotype, *M. luci* might have been misidentified as *M. ethiopica* in a number of surveys. Therefore, it is highly probable that this RKN has an even broader host range and distribution than is currently known. In December 2017, root galls with egg masses caused by a RKN were observed on the ornamental plant *Cordyline australis* (Forst f.) Hook. F (Ca) in Figueira da Foz, and the weed *Oxalis corniculata* L. (Oc), and tomato (Sl) in Montemor-o-Velho, all in Coimbra district. Egg masses were handpicked from infected roots of each plant and used to establish cultures of each isolate (Ca, Oc, and Sl) on tomato cv. Coração-de-Boi. Females were used to assess the esterase isoenzyme phenotype of each isolate to identify the species. The esterase phenotype from young egg-laying females protein extracts of the three isolates exhibited three bands of esterase activity (Rm: 0.38; 0.43; 0.48) (Fig. 1) corresponding to the *M. luci* L3 phenotype (Maleita et al., 2018). A *M. javanica* isolate (J3, Rm: 0.38; 0.45; 0.49) was used as reference isolate to determine the relative position of *M. luci* esterase bands (Fig. 1). Molecular identification was performed by amplification and sequencing of the cytochrome oxidase subunit I (COI) of mitochondrial DNA (mtDNA) region of one isolate of each location (Ca and Oc) using the primers JB3 (5′-TTTTTTGGGCATCCTGAGGTTTAT-3′) and 2R5 (5′-YTRWYCTTAAATCTAAATKMGTATG-3′) (Kiewnick et al., 2014). As no differences were observed between the sequences, the mtDNA region between cytochrome oxidase subunit II and 16S rRNA (mtDNA COII/16S rRNA) genes was only amplified and sequenced for Ca isolate using primers C2F3 (5′-GGTCAATGTTCAGAAATTTGTGG-3′) and MRH106 (5′-AATTTCTAAAGACTTTTCTTAGT-3′) (Maleita et al., 2018). DNA sequences were compared with available *Meloidogyne* species sequences in databases (Fig. 2). The length of all sequences of *Meloidogyne* spp. was set to 358 and 1,532 bp to mtDNA COI and mtDNA COII/16S rRNA regions, respectively, by removing several nucleotides to obtain a common start and end point. Phylogenetic analysis of mtDNA COI region was not very robust in differentiating *M. luci* from *M. ethiopica* (Fig. 2A), as also stated by Powers et al. (2018) for the most common RKN species (*M. arenaria*, *M. incognita*, and *M. javanica*). The Portuguese Oc and Ca sequences differed by only one nucleotide position from *M. ethiopica*, considering the 358 bp. *Meloidogyne luci* mtDNA COI sequence from Guatemala (KU372171.1) is similar to *M. ethiopica* (KU372162.1). On the other hand, the mtDNA COII/16S rRNA region differentiate these two closely related RKN species and proved to be useful for analyzing their relationship (Stare et al., 2017). Phylogenetic analysis revealed that mtDNA COII/16S rRNA sequences determined here formed a single cluster with all *M. luci* isolates (89% bootstrap), confirming the presence of *M. luci* (Fig. 2B). The mtDNA COII/16S rRNA sequence of Ca isolate and the other *M. luci* sequences were similar, with four to seven differences in alignment, while Ca *M. luci* sequence differed by 11 to 12 positions from *M. ethiopica* sequences. Sequences were submitted to GenBank database under the accession numbers MK190952 (isolate Ca) and MK190953 (isolate Oc) for mtDNA COI and MK190954 (isolate Ca) for mtDNA COII/16S rRNA genes. To our knowledge, this is the first report of *M. luci* infecting *C. australis* and *O. corniculata*. The detection of *M. luci* in two new locations (Figueira da Foz and Montemor-o-Velho) can be an indication that this nematode species could already be established and widespread in Portugal. Furthermore, these results draw attention to the importance of governmental inspections and the use of clean soil in nurseries. If ornamental plants, such as *C. australis*, were grown in infested soil, this will aid the transfer of plant-parasitic nematodes to new regions and/or other suitable hosts, with potential impact on economically important crops. Therefore, a survey for evaluation of *M. luci* distribution in Portugal is needed to decrease the risk of spread and to determine its potential economic impact.
